# Comparison of Two Cyberknife Planning Approaches for Multiple Brain Metastases

**DOI:** 10.3389/fonc.2022.797250

**Published:** 2022-02-03

**Authors:** Tianlong Ji, Yaowen Song, Xinyu Zhao, Yuzi Wang, Guang Li

**Affiliations:** Department of Radiation Oncology, The First Hospital of China Medical University, Shenyang, China

**Keywords:** Cyberknife, Iris collimator, treatment planning, stereotactic radiosurgery, multiple brain metastases

## Abstract

**Purpose:**

To compare the delivery efficiency, plan quality, and planned treatment volume (PTV) and normal brain dosimetry between different Cyberknife planning approaches for multiple brain metastases (MBM), and to evaluate the effects of the number of collimators on the related parameters.

**Methods:**

The study included 18 cases of MBM. The Cyberknife treatment plans were classified as Separate or Combined. For the Separate plan, each lesion was targeted by the collimator auto-selection method (Conformality 2/3 collimators). For the Combined plan, a PTV including all PTVs was targeted by the collimators. Monitor units (MUs), number of nodes and beams, estimated fraction treatment time (EFTT), new conformity index (nCI), dose gradient index (GI), homogeneity index (HI), PTV minimum/maximum dose (D_max_/D_min_), volume doses (D_2%_ and D_98%_), maximum doses to lenses, optic nerves, and brainstem as well as normal brain 3, 6, 10, and 12 Gy (*V*_3Gy_*–V*_12Gy_) were compared.

**Results:**

Compared to the Combined plan, the Separate plan had fewer nodes and beams, shorter EFTT, smaller PTV D_min_, normal brain dose, and GI, and larger HI. The Separate plan with 2 collimators also had worse PTV coverage. In the Combined plan, more collimators increased beams, EFTT, GI, and normal brain dose but improved the PTV D_min_. Among treatments based on the Separate approach, there were obvious differences between plans for most of the items except the nCI. Fewer collimators resulted in significantly reduced beams, EFTT, PTV D_98%_, and normal brain dose with improved GI, although PTV D_min_ and MUs were decreased while HI was increased.

**Conclusion:**

Both approaches met the requirements for SRS/HFSRT. We found that Separate plans improved treatment efficiency and normal tissue dosimetry.

## Introduction

Stereotactic radiosurgery (SRS) and hypo-fractionated stereotactic radiotherapy (HFSRT) are efficient and well-tolerated treatment modalities for patients with brain metastases (1, 2). In patients with multiple brain metastases (MBM), both techniques have proven effective for reducing neurotoxicity and preserving quality of life (3–5). SRS and HFSRT can be performed with a Gamma Knife (GK; Elekta AB; Stockholm, Sweden), a Cyberknife robotic radiosurgery system (CK; Accuray, Sunnyvale, CA, USA), or a conventional linear accelerator, and in the treatment of MBM, there may be marked differences in plan quality and delivery efficiency among these devices (6, 7).

The delivery and plan optimization features of the CK have been associated with excellent overall survival and local control rates in MBM, with relatively low toxicity (8). The CK has a 6-MV accelerator mounted on a robotic arm that is designed to deliver non-isocenter non-coplanar beam arrangements; this is combined with a high-resolution image-guided tracking system to help maximize the accuracy of the treatment. The positions of the nodes are fixed, and the beams vary according to the target.

**Figure 1:**
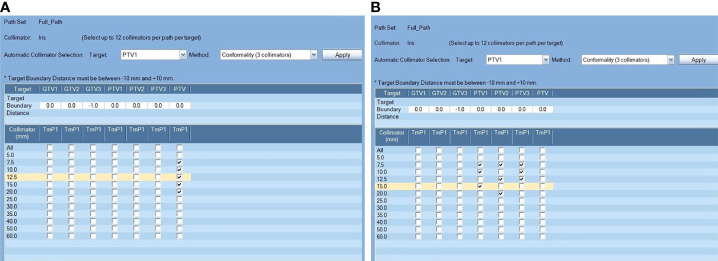
Comparison of two different treatment planning approaches. Lesions were targeted separately **(A)** or all of the lesions were included in a single PTV **(B)**; the “Conformality(3collimator)” auto-selection method was applied for the two planning approaches in this example.

Regardless of the equipment, different treatment planning approaches will lead to differences in plan quality and delivery efficiency (9, 10). The CK Model M6™ that was evaluated in this research is equipped with a fixed cones collimator as well as an Iris™ variable aperture collimator. The variable aperture collimator can provide better delivery efficiency than the fixed cone collimator by freely changing collimator sizes. Generally, the collimator selection is based on experience. It can also depend on the recommendations generated by a treatment planning system (TPS; e.g., Multiplan v5.3). **Figure 1** shows a TPS-generated collimator selection. The selection of collimators can affect the target coverage, the sparing of organs at risk (OARs), and the delivery efficiency (11).

Treatment planning for MBM is particularly complex because of the close proximity of the treatment targets to neighboring critical and radiosensitive structures (12). The number of contours, including the OARs and the target, is limited to 23 in the TPS, and the extension of each lesion to a separate planned treatment volume (PTV) is limited, especially in cases of more than 5 lesions. Because of this, a PTV including all lesions is sometimes used for the process of collimator selection, rather than basing the collimator selection on each lesion separately.

The purpose of this study was to compare CK treatment planning approaches for MBM in terms of the delivery efficiency, dosimetry of PTV and normal brain, and plan quality. In addition, for each approach, we compared two collimator selection methods with different numbers and sizes of Iris collimators in order to evaluate the effects of the number of collimators on the dosimetry.

## Methods

### Lesions

Eighteen patients treated for MBM with the CK Model M6™ were included in this study. Lesion numbers varied from two to six per patient, and a total of 73 lesions were enrolled (**Table 1**). Contour delineation was based on 1-mm CT and MRI slice thickness. The PTV was defined as the gross tumor volume (GTV) plus 1 mm for all plans. Each GTV was extended separately to generate a PTV, and a PTV_all_ including all PTVs was then generated for this analysis. The OARs included skin, normal brain, eyes, lenses, brainstem, cochlea, and optic nerves. The normal brain area was defined as the whole brain minus PTV_all_. Detailed tumor information is presented in **Table 1**.

**Table 1:** Detailed tumor information.

Item		Value
Number of patients		18
Number of lesions:	Total	73
	Median	4
Volume of lesions (cm^3^):	Min	0.409
	Max	26.645
	Average for lesions	5.837
	Average for cases	23.828

### Treatment Plans

Four plans were made for each case according to either the Separate or the Combined approach, as described below. The prescription dose was 35 Gy/5 F for all cases. The beam intersection model was set to exclude the eyes and the brainstem. The optimization goal for the PTV was set as optimized minimum dose (OMI) equal to the prescription dose. All treatment plans implemented time reduction (70% the time evaluated at first optimization) and were normalized at 95% PTV covered by 100% prescription dose.

For the Separate approach (Sep), PTVs were selected separately as the target in the model of collimator selection (**Figure 1A**). For the Combined approach (All), PTV_all_ was selected as the target in the model of collimator selection. To pick the correct collimator size, conformality(2or3 collimator) auto-selection methods with 2 and 3 collimators were applied for each approach (**Figure 1**). Accordingly, the corresponding treatment plans for each approach are named Sep_2 and Sep_3 and All_2 and All_3.

### Plan Evaluation

The plans were evaluated for monitor units (MUs); number of nodes and beams; estimated fraction treatment time (EFTT); new conformity index (nCI); dose gradient index (GI); homogeneity index (HI); dose-volume histogram (DVH) parameters for PTV including minimum dose (D_min_) and maximum dose (D_max_); the 2% PTV maximum dose (D_2%_); and the 98% PTV minimum dose (D_98%_). For normal brain tissue, parameters including volumes receiving a specific dose at 3, 6, 10, and 12 Gy (V_3Gy_, V_6Gy_, V_10Gy_, V_12Gy_) were evaluated. Maximum doses to lenses, optic nerves, and brainstem were also evaluated. The optic chiasm is included with the same dose limitation setting in our department.

The EFTT was evaluated by the TPS according to the number of nodes, beams, and MUs along with a user-defined estimated patient setup time and estimated image time interval.

The nCI was used to evaluate the conformity of the dose distribution and calculated as: nCI = (V_t_ * V_p_)/(V_tp_)^2^, where V_tp_ is the PTV within the prescription isodose surface, V_t_ is the PTV, and V_p_ is the prescription volume (13).

HI indicates the degree of uniformity of dose distribution within the target and was calculated as: HI = (max dose/prescribed dose).

GI describes the steepness of the dose gradient and was calculated as: GI = V_50%_D_p_/V_p_, where V_50%_D_p_ is 50% of the prescription isodose line volume and V_p_ is the prescription volume.

### Statistical Analysis

All statistical analyses were performed with SPSS 20.0 (SPSS Inc., Chicago, IL, USA). All data were normalized by parametric tests to accommodate comparison by paired samples t-test. The Wilcoxon signed-rank test was considered the non-parametric counterpart of the paired t-test. *p* < 0.05 was considered as statistically significant.

## Results

The treatment delivery efficiency parameters are shown in **Table 2**. Compared to the Combined plans, the Separate plans had fewer nodes (142.4 vs. 151.6 and 153.2 vs. 156.0; *p* = 0.011 and <0.001) and beams (379.6 vs. 437.9 and 464.3 vs. 510.1, *p <*0.001) and shorter EFTTs (58.8 min vs. 63.7 min and 65.3 min vs. 68.9 min, *p* < 0.001 and 0.005). There were no significant differences for MUs.

**Table 2:** Treatment delivery efficiency: MUs, nodes, beams, and EFTT (minutes) among the four plans.

	ALL_2	ALL_3	Sep_2	Sep_3	ALL_2 VS ALL_3	Sep_2 VS Sept_3	ALL_2 VS Sep_2	ALL_3 VS Sep_3
MUs	54,497.6 ± 6410.9	53,835.6 ± 6788.4	55,910.5 ± 7985.7	54,446.7 ± 6886.0	0.022*^a^*	0.016*^b^*	0.064*^b^*	0.107^b^
Nodes	151.6 ± 17.3	156.0 ± 11.6	142.4 ± 19.1	153.2 ± 12.6	0.011*^b^*	<0.001*^a^*	0.001*^a^*	0.019^a^
Beams	437.9 ± 142.7	510.1 ± 168.4	379.6 ± 127.8	464.3 ± 145.8	<0.001*^a^*	<0.001*^a^*	<0.001*^b^*	0.005^b^
EFTT (min)	63.7 ± 18.6	68.9 ± 20.6	58.8 ± 17.9	65.3 ± 19.0	<0.001*^a^*	<0.001*^a^*	<0.001*^b^*	0.005^a^

a Comparison by paired samples t-test.

b Comparison by Wilcoxon signed-rank test.

For the same approach with different collimator settings, smaller collimator values resulted in increased MUs (54,497.6 vs. 53,835.6 and 55,910.5 vs. 54,446.7, *p* = 0.022 and 0.016) with fewer beams (437.9 vs. 510.1 and 379.6 vs. 464.3, *p* < 0.001) and shorter EFTTs (63.7 min vs. 68.9 min and 58.8 min vs. 65.3 min, *p* < 0.001) and fewer nodes (151.6 vs. 156.0 and 142.4 vs. 153.2, *p* = 0.011 and 0.001) in the Separate approach. There were no significant differences for the other metrics.

The Separate approach produced obviously lower PTV D_min_ values (3132.1 cGy vs. 3015.6 cGy and 3197.8 cGy vs. 3149.5 cGy, *p* = 0.010 and 0.026) compared to the Combined approach. For treatment plans with 2 collimators, the Combined approach produced lower PTV D_max_ (4919.5 cGy vs. 5101.1 cGy, *p* = 0.020) and D_2%_ (4581.1 cGy vs. 4788.1 cGy, *p* = 0.003) values, respectively.

The plans with two collimators using the Separate approach generated greater PTV D_max_ (5101.1 cGy vs. 4860.9 cGy, *p* = 0.020) and D_2%_ (4788.1 cGy vs. 4528.5 cGy, *p* = 0.001) values. Meanwhile, fewer collimators resulted in lower PTV D_min_ values (3132.1 cGy vs. 3197.8 cGy and 3015.6 cGy vs. 3149.5 cGy, *p* = 0.005 and 0.002) for plans on both approaches. There were no significant differences for the other metrics. The details are shown in **Table 3**.

**Table 3:** DVH parameters for PTV among the four plans.

	ALL_2	ALL_3	Sep_2	Sep_3	ALL_2 VS ALL_3	Sep_2 VS Sept_3	ALL_2 VS Sep_2	ALL_3 VS Sep_3
Dmin (cGy)	3132.1 ± 105.1	3197.8 ± 80.3	3015.6 ± 152.7	3149.5 ± 91.0	0.005*^a^*	0.002*^a^*	0.010*^a^*	0.026*^a^*
Dmax (cGy)	4919.5 ± 296.4	4741.2 ± 238.6	5101.1 ± 403.9	4860.9 ± 276.0	0.172*^a^*	0.001*^b^*	0.020*^b^*	0.208*^a^*
D_2%_ (cGy)	4581.1 ± 40.2	4472.4 ± 236.1	4788.1 ± 330.6	4528.5 ± 275.0	0.084*^a^*	0.001*^a^*	0.003*^b^*	0.106*^a^*
D_98%_ (cGy)	3465.9 ± 32.0	3472.5 ± 24.2	3442.0 ± 28.9	3463.7 ± 25.6	0.378*^a^*	0.017*^a^*	0.015*^a^*	0.263*^a^*

a Comparison by paired samples t-test.

b Comparison by Wilcoxon signed-rank test.

There were very clear differences among the plans for the protection of normal brain, and for the same number of collimators, the Separate approach produced lower normal brain doses. By the same approach, the fewer the collimators, the lower the dosimetry for normal brain.

These results are shown in **Table 4**.

**Table 4:** Dosimetric results for normal brain tissue, lens, optical nerves, and brainstem.

	ALL_2	ALL_3	Sep_2	Sep_3	ALL_2 VS ALL_3	Sep_2 VS Sept_3	ALL_2 VS Sep_2	ALL_3 VS Sep_3
V12Gy	169.5 ± 88.2	192.4 ± 98.6	151.7 ± 78.0	172.4 ± 80.0	<0.001*^b^*	<0.001*^b^*	<0.001*^b^*	<0.001*^b^*
V10Gy	220.3 ± 119.3	252.8 ± 134.7	193.6 ± 100.0	222.5 ± 103.8	<0.001*^b^*	<0.001a	<0.001*^b^*	0.001*^b^*
V6Gy	439.4 ± 213.6	517.3 ± 237.0	386.8 ± 180.0	450.4 ± 182.0	<0.001*^b^*	<0.001a	0.002*^b^*	0.001*^b^*
V3Gy	826.7 ± 257.2	925.4 ± 253.9	733.8 ± 243.2	861.2 ± 238.4	<0.001*^a^*	<0.001*^a^*	<0.001*^b^*	0.001*^a^*
Lens	49.3 ± 44.8	56.2 ± 21.9	48.7 ± 38.3	62.4 ± 34.5	0.237*^b^*	0.398*^b^*	0.871*^b^*	0.310*^b^*
Nerves	268.9 ± 205.9	370.9 ± 282.1	303.9 ± 230.4	337.3 ± 244.9	0.091*^b^*	0.499*^b^*	0.866*^b^*	0.735*^b^*
Brainstem	546.5 ± 502.6	615.9 ± 466.7	515.4 ± 380.2	560.5 ± 447.3	0.028*^b^*	0.499*^b^*	0.612*^b^*	0.091*^b^*

a Comparison by paired samples t-test.

b Comparison by Wilcoxon signed-rank test.

As shown in **Table 4**, for the same number of collimators, the plans on the Separate approach showed obviously increased HI (1.46 vs. 1.41 and 1.37 vs. 1.36, *p* = 0.016 and 0.016) and decreased GI (4.10 vs. 4.49 and 4.75 vs. 5.06, *p* = 0.014 and 0.001) in comparison with the Combined approach. There were no significant differences in the nCI.

The plans with two collimators showed increased HI (1.41 vs. 1.36 and 1.46 vs. 1.37 *p* = 0.016 and 0.001). Meanwhile, fewer collimators resulted in better GI (4.49 vs. 5.06 and 4.10 vs. 4.75, *p* < 0.001) for plans on both approaches. There were no significant differences for the other metrics. The details are shown in **Table 5**.

**Table 5:** Dosimetric results for plan quality.

	ALL_2	ALL_3	Sep_2	Sep_3	ALL_2 VS ALL_3	Sep_2 VS Sept_3	ALL_2 VS Sep_2	ALL_3 VS Sep_3
nCI	1.19 ± 0.08	1.19 ± 0.07	1.22 ± 0.08	1.15 ± 0.08	0.143*^a^*	0.072*^a^*	0.406*^b^*	0.263*^a^*
HI	1.41 ± 0.08	1.36 ± 0.07	1.46 ± 0.12	1.37 ± 0.08	0.016*^a^*	0.001*^b^*	0.016*^b^*	0.016*^a^*
GI	4.49 ± 0.77	5.06 ± 0.95	4.10 ± 0.60	4.75 ± 0.85	<0.001*^b^*	<0.001*^a^*	0.014*^a^*	<0.001*^b^*

a Comparison by paired samples t-test.

b Comparison by Wilcoxon signed-rank test.

## Discussion

Depending on lesion numbers and proximity of lesions to vital and radiosensitive structures, separate or combined PTVs can be defined for CK treatment planning in MBM. This study compared separate and combined planning approaches with the same optimization parameters for quality, delivery efficiency, and PTV and normal brain dosimetry and examined the impact of the number of collimators on the plans with different approaches.

**Figure 2:**
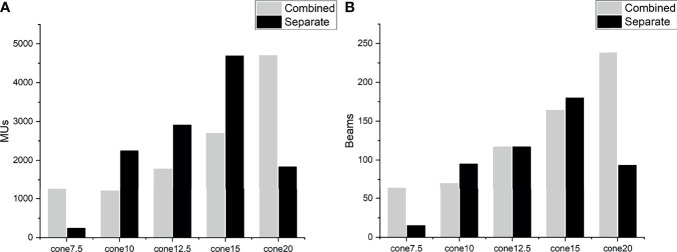
Comparison of MUs **(A)** and beams **(B)** in different optimization approaches.

There were significant differences between the planning approaches. With the same collimator selection, the separate approaches resulted in fewer beams and nodes, shorter EFTTs, decreased doses to normal brain, and lower GI than the combined approaches, although the numbers were a little worse for PTV D_min_ and HI. A separate approach with two collimators also increased the PTV D_max_ and D_2%_ and decreased the PTV D_98%_. This might be explained by the characteristics of the optimization algorithm. Schlaefer et al. (14) presented a stepwise optimization algorithm to optimize multiple clinical goals in steps with built-in priority. This was carried out in the CK planning system as the sequential optimizer. However, this algorithm cannot identify the spatial relationship between PTVs because all PTVs are projected onto one image in the process of optimization. The TPS optimizes the PTVs as a target that is irregular in shape and spatial position. Therefore, plans that combine PTVs may generate more and larger Iris collimators. Planning with combined PTVs resulted in more minimum and maximum sizes of the Iris collimators and the same appearance of MUs (**Figure 2**). A few studies have investigated the relationship of collimator diameter and the target coverage and have found that a larger collimator can reduce dose uncertainty and improve target coverage (15, 16). In this study, we found that planning for separate PTVs resulted in a slightly decreased minimum PTV dose. On the other hand, more beams through two PTVs introduced a higher dose to the space between the PTVs, which is why the normal brain dose is so high for the combined plans.

We also evaluated the effect of the number of collimators on the plan quality, delivery efficiency, and dosimetry with different optimization approaches. There was a significant difference in the number of nodes between the plans. Actually, the number of collimators per target may exceed 2 or 3 for the combined PTVs. Therefore, the two approaches demonstrated different effects. For the combined PTV approach, there were no significant differences in PTV D_max_, D_2%_, D_98%_, and nCI, which indicates that too many collimators might actually hinder improvement of the plan quality. More collimators also obviously increased nodes, beams, EFTT, GI, and dose to normal brain and decreased the MUs and HI, although improved PTV coverage produced slightly higher PTV D_min_. These results are consistent with those of Varnava et al., who conjectured that 2 collimators can do the same as the 10 collimators (17).

When planning for the separate PTVs, there were obvious differences between the plans with different numbers of collimators for most of the items, except the nCI. The plans with two collimators allowed significant reduction in the dose to normal tissue, with corresponding GI improvement, while at the same time beams and EFTT were also reduced. With three collimators, the PTV coverage was improved by increasing the PTV D_min_ and D_98%_ and decreasing the PTV Dmax, D_2%_, and HI. These findings agreed with the data of Fuller et al., who demonstrated that multiple collimator sizes improved the plan quality (18). However, a similar plan quality can be obtained by using three collimator sizes, instead of all 12, with a smaller incremental increase (19). Our results indicated that in planning for separate PTVs, two Iris collimators can provide similar quality to three collimators. It is critical to consider not only the conformity and homogeneity of the PTV but also the protection of the OARs, because increasing the number of Iris collimators usually results in limited improvement of dose coverage for PTV and plan quality (HI) with longer treatment times but worsens the dosimetry of normal brain. Therefore, the collimator number should be adjusted to allow a good balance between the treatment target and the OARs.

Since we knew that different optimization strategies and object function parameters would affect the plans across many results, all cases in this study were normalized for consistency parameters. However, according to our experience, when the dose volume lower limit (DVL) is used to optimize the PTV, the treatment time will be significantly increased, but this has the same effect for either planning approach. However, we cannot guarantee that the two methods increase the time in the same proportion, and this is one of the limitations of this study. In addition, the lens and brainstem were avoided at beam intersections, which meant that the dose of these OARs was not affected by the planning approaches. This also indirectly shows that the real differences between the two approaches are the difference in the selection of collimators and the difference in the penetration path of the beam on the target.

Although planning for separate lesions clearly showed better performance for delivery efficiency, normal tissue sparing, and plan quality, the total number of delineable organs (including targets and OARs) in the TPS was limited. Therefore, when there are more than six lesions, some workarounds that do not impact the quality of the plan can be applied, such as using both eyes, rather than each eye separately, so that there can be one more target. On the other hand, planning for separate PTVs is not suitable for cases where there are more OARs and lesions that need to be clearly defined.

There were several more limitations of this study. The EFTTs were overestimated due to the lack of time reduction, and other OARs, including the lens, brainstem, and nerves, were not evaluated because the dose was far below the limitation with two approaches by the avoidance of setting in the model of beam intersection.

## Conclusion

Both approaches can achieve high-quality treatment plans for SRS/HFSRT in MBM. The Separate approach improved treatment efficiency and dose for normal tissues. The increased number of collimators with the Separate approach decreased treatment efficiency and increased brain dose but improved PTV coverage and plan quality. The selection of the number of collimators must balance PTV coverage and normal tissue sparing.

## Data Availability Statement

The original contributions presented in the study are included in the article/supplementary material. Further inquiries can be directed to the corresponding author.

## Author Contributions

TJ designed the study. YS, XZ, YW, and GL collected the patients’ clinical data and delineated the target volume and reviewed the patients’ treatment plans. TJ analyzed the data and wrote the paper. All authors contributed to the article and approved the submitted version.

## Conflict of Interest

The authors declare that the research was conducted in the absence of any commercial or financial relationships that could be construed as a potential conflict of interest.

## Publisher’s Note

All claims expressed in this article are solely those of the authors and do not necessarily represent those of their affiliated organizations, or those of the publisher, the editors and the reviewers. Any product that may be evaluated in this article, or claim that may be made by its manufacturer, is not guaranteed or endorsed by the publisher.
